# Prognostic Role of Inflammasome Components in Human Colorectal Cancer

**DOI:** 10.3390/cancers12123500

**Published:** 2020-11-24

**Authors:** Charlotte Domblides, Isabelle Soubeyran, Lydia Lartigue, Isabelle Mahouche, Félix Lefort, Valérie Velasco, Thomas Barnetche, Patrick Blanco, Julie Déchanet-Merville, Benjamin Faustin

**Affiliations:** 1 ImmunoConcEpt Laboratory, CNRS UMR 5164, Bordeaux University, 33076 Bordeaux, France; felix.lefort@chu-bordeaux.fr (F.L.); patrick.blanco@chu-bordeaux.fr (P.B.); jdechanet@immuconcept.org (J.D.-M.); 2 Department of Medical Oncology, Hôpital Saint-André, Bordeaux University Hospital-CHU, 33000 Bordeaux, France; 3 Comprehensive Cancer Center, Department of Biopathology, Institut Bergonié, 33000 Bordeaux, France; i.soubeyran@bordeaux.unicancer.fr (I.S.); v.velasco@bordeaux.unicancer.fr (V.V.); 4 INSERM, U1218 ACTION, F-33000 Bordeaux, France; lydia.lartigue@gmail.com (L.L.); i.mahouche@bordeaux.unicancer.fr (I.M.); 5 Department of Rheumatology, Bordeaux University Hospital, 33000 Bordeaux, France; thomas.barnetche@chu-bordeaux.fr; 6 Department of Immunology and Immunogenetic, FHU ACRONIM, Bordeaux University Hospital, 33000 Bordeaux, France; 7 Immunology Discovery, Janssen Research and Development, 3210 Merryfield Row, San Diego, CA 92121, USA

**Keywords:** colorectal cancer, inflammasome, prognostic, immune infiltration

## Abstract

**Simple Summary:**

Inflammasomes are critically involved in gut epithelial homeostasis, immunosurveillance and in controlling tumorigenesis mechanisms. Data on the role of inflammasomes in tumorigenesis are mostly provided by transcriptomic analyses of bulk tumors, eluding a potential specific role of intrinsic epithelial inflammasomes. Therefore, we investigated the expression of inflammasome components in intestinal epithelial cells, at the protein level in patient tissues and assessed the correlation with clinicopathological parameters. We found that downregulation of the epithelial expression of NOD-like receptor family pyrin domain containing 6 (NLRP6) and IL-18 was associated with more advanced disease and worse patients’ outcome. Furthermore, the loss of both epithelial and stromal IL-18 was also associated with worse disease outcome. Finally, we identified an epithelial innate immune protein profile combining NLRP6 and IL-18 that stratified patients for better clinical prognosis. Together, analysis of epithelial inflammasomes may help clinical decisions for better prognostic assessment and may identify new therapeutic targets in colorectal cancer.

**Abstract:**

(1) We wanted to assess the prognostic impact of inflammasomes involved in gut epithelial homeostasis and the development of human colorectal cancer (CRC). (2) We investigated the expression of inflammasome components in colonic epithelial cells at the protein level in patient tissues, through an immunofluorescence assay. (3) In a cohort of 104 patients, we found that all inflammasome components were downregulated in CRC. Loss of epithelial (but not stromal) expression of NLRP6, caspase-1 and IL-18 was associated with an increased mortality of 72%, 58% and 68% respectively and to disease progression into metastasis. The loss of epithelial and stromal IL-18 but not NLRP6, was associated to lower tumor immune infiltrates in the lymphoid compartment and higher Programmed cell Death receptor 1 (PD-1) expression. Finally, we found that combined downregulation of IL-18 and NLRP6 was associated with a worse outcome. Indeed, 5-year survival rates were 26% for the NLRP6low/IL-18low tumors, compared to 64.4% for the entire cohort. This downregulation was associated with a more advanced disease (*p* < 0.0001) and a trend to lower lymphoid cell infiltration. (4) We identified critical inflammasome markers that may help in better stratifying patients for prognosis in CRC and could help clinicians to determine which patients may benefit from immunotherapies.

## 1. Introduction

Immunotherapy is one of the most promising approaches for the treatment of several types of cancer. Indeed, prolonged survival rates were observed with immune checkpoint inhibitors that reactivate immune system antitumor functions. However, 60% to 80% of patients are resistant to these treatments [1,2] and there is a crucial need to identify new therapeutic targets to improve patient outcome.

Colorectal cancer (CRC) is one of these refractory tumors, with response rates of 30-40% in mismatch repair-deficient or microsatellite instability-high diseases and under 10% in the absence of these abnormalities [3]. CRC is the third most common cancer worldwide and the second in terms of mortality [4]. Colorectal carcinogenesis is highly correlated to chronic harmful inflammation and to gut microbiome dysbiosis [5]. Tumor-mediated inflammation is an emerging hallmark of cancer which is tightly controlled. Indeed, chronic and uncontrolled inflammation favors intestinal epithelial lesions which, among other mechanisms, induces carcinogenesis. On the other hand, innate immune mechanisms are required to impair early tumorigenesis by priming a robust antitumor immune response mediated by myeloid and lymphoid cells. 

The colon is the predominant site for host: microbiome interactions. To preserve host homeostasis and avoid uncontrolled inflammation, these bacteria are tightly controlled through their recognition by pattern-recognition receptors (PRRs). Nucleotide-binding domain and leucine-rich repeat proteins (NLRs) are PRRs emerging as players of host homeostasis. There are 22 NLRs described in human but only some of them are able to form an inflammasome platform [6]. Inflammasomes are multi-protein macromolecular cytosolic complexes formed after recognition by NLRs of specific danger- or pathogen-associated, molecular patterns (respectively DAMPs or PAMPs). Once activated, NLRs bind to the adaptor protein ASC (Apoptosis-associated speck like protein containing a caspase recruitment domain) and pro-caspase-1. This leads to the cleavage and activation of caspase-1 which in turn maturates IL-1β and IL-18 for secretion. Multiple inflammasomes are described in hematopoietic cells (such as dendritic cells, macrophages). However, it also exists in epithelial cells where it plays an important role in tissue homeostasis through recovery after tissue damage and defense against external insults, limiting chronic inflammatory processes. 

Inflammasomes are critically involved in maintaining gut epithelial homeostasis but also play a crucial role in immunosurveillance and tumorigenesis mechanisms [7]. Indeed, in human, a transcriptomic study has found that the expressions of NLRP1 (NOD-like receptor family pyrin domain containing 1), NLRP3, NLRC4 (NLR Family CARD Domain Containing 4 ) and AIM2 (Absent in Melanoma 2) were reduced in Chinese patients with CRC [8]. The antitumor functions of these NLRs seemed to be partly due to IL-18 secretion in the mucosa and its loss was shown to be associated with carcinogenesis in different mouse models [9]. However, it was recently reported that inflammasome components may deploy antitumor functions in a caspase-independent manner. Indeed, AIM2 was associated with CRC in mouse models through the decrease of AKT activation [10]. NLRP12 is also able to regulate NF-κB, MAPK and AKT and its loss in colon epithelium was associated with higher tumorigenesis in mouse models [11]. In most studies, interpretations of the role of inflammasomes in cancer have relied on transcriptome analyses of bulk tumors and their specific tumorigenic role in various mucosal cell types is unclear. Here, we further investigated the expression of various inflammasome components in intestinal epithelial cells, at the protein level in patient tissues and identified their prognostic impact in the development of human CRC.

## 2. Results

### 2.1. Population Characteristics

Clinical features of patients from Bergonié Institute are provided in Table 1. Patients were predominantly men (57.7%) with a median age of 71.7 [range: 38.6–88.4] years. Rectal cancers represented 16.3% of our cohort (*n* = 17). Colorectal cancers were mainly stage II (34.6%) and III (35.6%), with predominant grade 2 (73.1%). In our cohort, 15.4% of tumors had a microsatellite instable (MSI) status. Diagnosis was obtained by surgery (97.1%) for almost all patients. After surgery, 40.4% patients did not receive any adjuvant therapy because of local diseases and 45.2% received an adjuvant chemotherapy due to locally advanced disease. Furthermore, 13.5% received palliative chemotherapy because of advanced diseases. After a median follow-up of 62.1 months, median overall survival was not reached and 5-years survival was 64.4%. 

### 2.2. Correlation of the Expression of Epithelial Tumour Inflammasome Components with Clinical Outcomes

**Figure 1:**
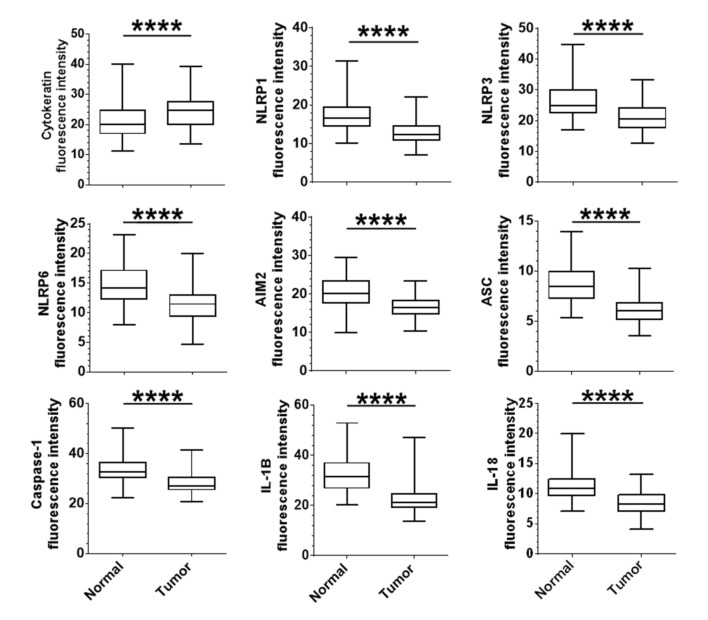
Epithelial protein expression of inflammasome components are downregulated in colorectal cancer patients: Protein expression of cytokeratin, inflammasome components and cytokines in colorectal epithelial normal and tumor cells. Expression levels of each marker were assessed as the mean of all median fluorescence intensity in each cell contained in the cytokeratin mask for each sample of normal and tumor tissues. The mean intensity value of the 3 tumor spots was compared to the mean intensity value of the 2 normal matched samples for each patient. Paired t-test (parametric) or Wilcoxon matched-pairs (non-parametric) were used to evaluate the significance of the difference between normal and tumor tissue. **** *p* < 0.0001.

We first assessed the immunofluorescence intensity of selected inflammasome components (NLRP1, NLRP3, NLRP6, AIM2, ASC, Caspase-1, IL-1β and IL-18) in tumor cells compared to normal epithelial cells, for each patient. As show in Figure S1, slides were stained using a specific antibody against each component of the inflammasome together with an antibody against cytokeratin (an epithelial marker) and DAPI (a nuclear marker). Images analysis was then done as follows (for details, see Supplementary Methods). First, nuclear (DAPI positive) and cellular segmentation was performed. Then, a cytokeratin mask was created from the cytokeratin positive segmented cells and superimposed on the inflammasome marker staining. Finally, expression quantification of each inflammasome marker was obtained by measuring the fluorescence intensity of DAPI positive cells in the cytokeratin mask in each spot. Using this tissue-based approach, we found a significant downregulation at the protein level of all inflammasome components in epithelial tumor cells (*p* < 0.001) (Figure 1). Conversely, cytokeratin expression in epithelial tumor cells was higher than in normal epithelial cells. 

**Figure 2:**
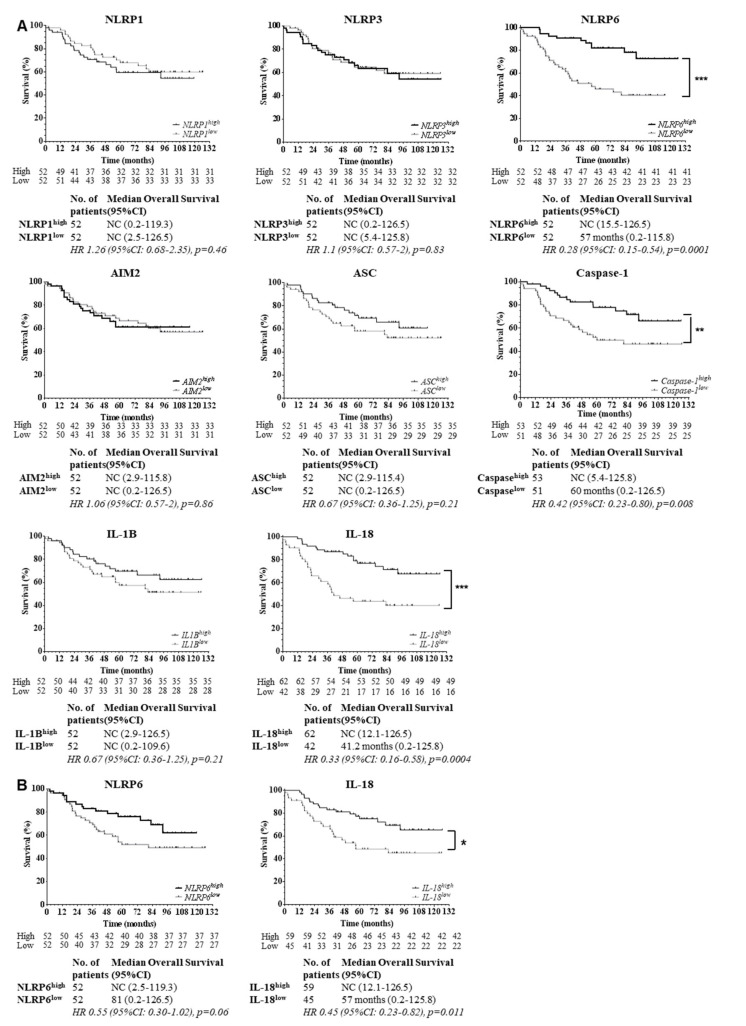
NLRP6 and IL-18 expressions in epithelial tumor cells are strong predictors of patients’ outcome. (**A**) NLRP6, caspase-1 and IL-18 were correlated with patients’ outcome in colorectal cancer. Survival curves of patients based on expression of inflammasome proteins in epithelial tumor cells are shown. Patients were stratified by high or low expressions according to cut-offs predetermined with ROC curves (NLRP6, caspase-1 and IL-18) or the median of fluorescence intensity (NLRP1, NLRP3, AIM2, ASC, IL-1B). Censored data are indicated by tick marks. Data for any patients who were not known to have died at the time of the analysis were censored at the last recorded date that the patient was known to be alive. 95%CI: 95% confidence interval; HR: Hazard Ratio. A log-rank test stratified according to protein expressions was used. NC: could not be calculated. (**B**) Stromal protein expression of NLRP6 and IL-18 were not correlated to survival. Survival curves of patients according to NLRP6 and IL-18 expression in stromal cells from colorectal tumors are shown. Patients were stratified according to the median fluorescence intensity for each marker, by high or low expression, according to cut-offs determined with ROC curves. Censored data are indicated by tick marks. Data for any patients who were not known to have died at the time of the analysis were censored at the last recorded date that the patient was known to be alive. 95%CI: 95% confidence interval; HR: Hazard Ratio. A log-rank test stratified according to protein expressions was used. NC: could not be calculated.

We then assessed the correlation between expression of inflammasome components and overall survival. Interestingly, the expression of three biomarkers was associated with overall survival in our cohort: NLRP6, caspase-1 and IL-18 (Figure 2A). Indeed, we found that the downregulation, in epithelial tumor cells, of each biomarker was correlated with a poor prognosis, as demonstrated by an increased mortality rate of 72%, 58% and 68%, respectively. All other components were not correlated to patient outcome. Furthermore, in multivariate Cox regression, downregulation of NLRP6 and IL-18 in the epithelium was associated with a worse outcome, after adjustment on TNM tumor stage (NLRP6: OR 3.25 [95CI: 1.27–8.28], *p* = 0.013; IL-18: OR 2.6 [95CI: 1.02–6.63], *p* = 0.045). Hence, the protein downregulation of NLRP6, caspase-1 or IL-18 in epithelial tumor cells was correlated with a poor clinical outcome for colorectal cancer patients.

Because immune cells also express inflammasome components, we assessed the impact of NLRP6 and IL-18 stromal expression on overall survival (Figure 2B). We found a trend towards a worse outcome in stromal NLRP6low cohort but it did not reached significance (*p* = 0.06). Conversely, for IL-18, there was an association between low IL-18 production by stromal cells and worse outcome (*p* = 0.011). However, this correlation was not as important as the prognostic impact of IL-18 expression in epithelial tumor cells. Hence, stromal expression of NLRP6 and IL-18 was not associated with patients’ outcome to the same extent than their epithelial expression.

### 2.3. Correlation between Expression of Inflammasome Components and Clinical Parameters

We found higher expression of NLRP1, NLRP3 and IL-1β in the young subgroup of patients (≤67 years) in our cohort (respectively *p* = 0.03, *p* = 0.001 and *p* = 0.0002). There was no correlation between inflammasome expression and other clinical parameters. However, there was a trend to higher IL-18 epithelial expression in tumors with an MSI status (*p* = 0.057) and MSI status was also correlated to lower AIM2 expression (*p* = 0.015). Interestingly, stromal IL-18 expression was also high in MSI tumors (*p* = 0.0019) but not stromal NLRP6 (Figure S2A).

**Figure 3:**
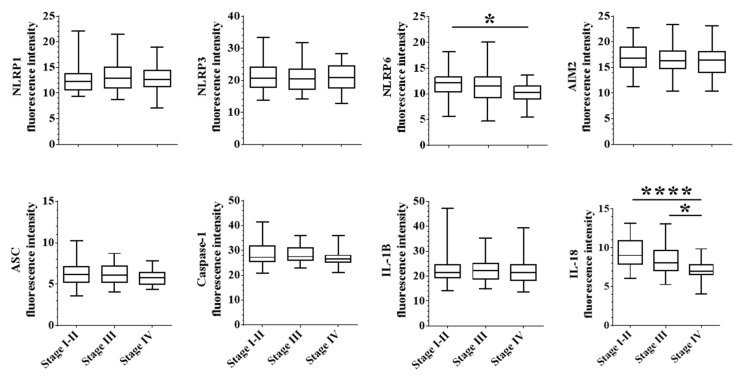
Epithelial expression of NLRP6 and IL-18 are correlated with tumor evolution. For each protein, we assessed the correlation between fluorescence intensity and tumor stages, classified in stage I-II (locally advanced), stage III (regionally advanced) and stage IV (metastatic disease). A one-way ANOVA (parametric) or Kruskal-Wallis (unparametric) tests were used to evaluate the significance of the differential expression between each disease stage. * *p* < 0.05 and **** *p* < 0.0001.

Then, we analyzed the association between epithelial inflammasome expression and TNM stage. We found that the downregulation of NLRP6 or IL-18 in epithelial tumor cells was associated with a more advanced disease (Figure 3). The effect of epithelial IL-18 downregulation seemed to be stronger on tumor evolution than epithelial NLRP6. Furthermore, we found that epithelial and to a lesser extent stromal expression of IL-18, was associated with a more advanced disease (*p* < 0.0001 and *p* = 0.0002, respectively) (Figure S2A). We did not find any correlation between stromal NLRP6 expression and TNM stage. Hence, the loss of NLRP6 or IL-18 in epithelial tumor cells was associated to metastatic progression and epithelial expression of inflammasome components had a more important impact on tumor evolution.

### 2.4. Correlation between Epithelial Expression of Inflammasome Components and Immune Infiltration

**Figure 4:**
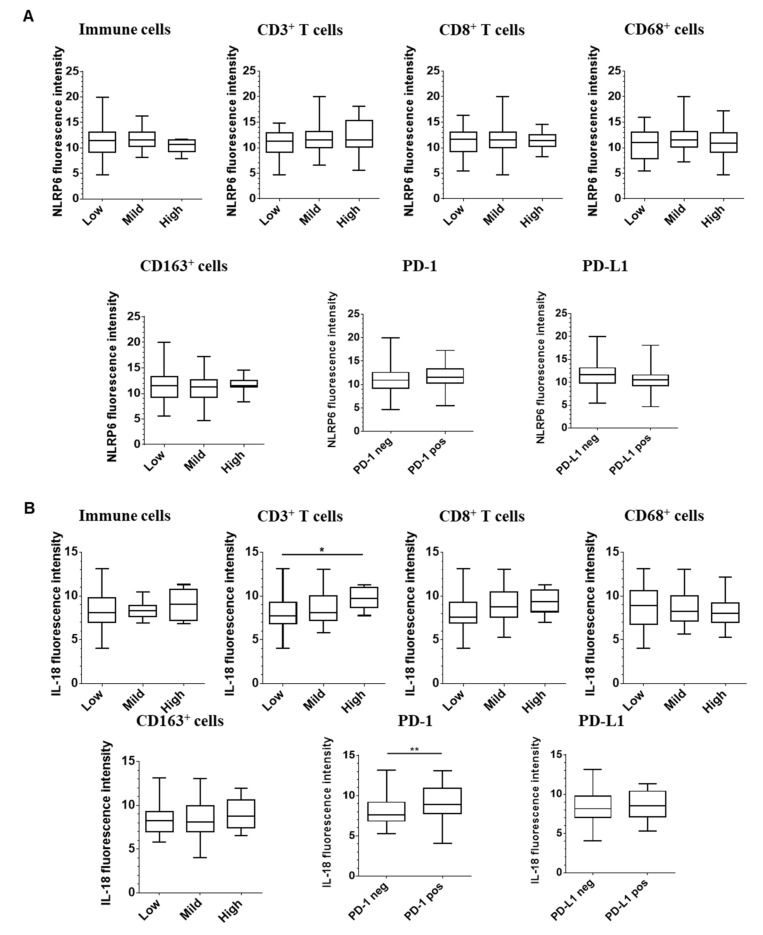
Expression of epithelial IL-18 is correlated with higher lymphocyte infiltration within colorectal tumors. For each protein, we assessed the correlation between fluorescence intensity of inflammasome component NLRP6 (**A**) or IL-18 (**B**) and immune infiltration assessed by immunohistochemistry for T cell lymphocytes (CD3+ and CD8+) and macrophages (CD68+ and CD163+). We also evaluated PD-1 and PD-L1 expressions. A one-way ANOVA (parametric) or a Kruskal-Wallis test (non-parametric) were used to evaluate the correlation between expression intensity and immune infiltrate levels. * *p* < 0.05 and ** *p* < 0.01.

We evaluated the association between immune infiltrates and expression of inflammasome components (Figure 4 and Figure S3), including overall immune infiltrate, CD3+ T cells, CD8+ T cells, CD68+ macrophages, CD163+ macrophages and expression of PD-1 and PD-L1. No correlation was observed between NLRP1, NLRP3, NLRP6, AIM2, caspase-1 or IL-1β expression and immune infiltration. However, high expression of ASC adaptor was associated with higher CD8+ T cell infiltration (*p* = 0.01) and higher PD-1 expression by immune cells (*p* = 0.02). Furthermore, tumors with low IL-18 epithelial expression were associated with low CD3+ T cell infiltration. There was also a trend to a lower CD8+ T cell infiltration in this group, suggesting that there are probably different sub-types of T cells involved. Finally, tumors with high levels of IL-18 were associated with higher expression of PD-1 receptor (*p* = 0.018). No association with macrophage infiltrate was observed. Hence, loss of IL-18 epithelial expression was associated with modification of immune cell infiltration, especially lymphoid cells.

Then, we assessed the association between stromal expression of NLRP6 or IL-18 and immune infiltration (Figure S2B). No correlation was observed between stromal expression of NLRP6 and immune cell infiltration and only an association between lower stromal NLRP6 expression and lower PD-1 expression by immune cells. However, we found a strong correlation between stromal IL-18 expression and CD3+/CD8+ T cell infiltration and PD-1. Hence, stromal expression of IL-18 is tightly linked to lymphoid immune infiltration and a marker of immune exhaustion.

### 2.5. The Role of the Inflammasome Protein Profile in Clinical Outcome

**Figure 5:**
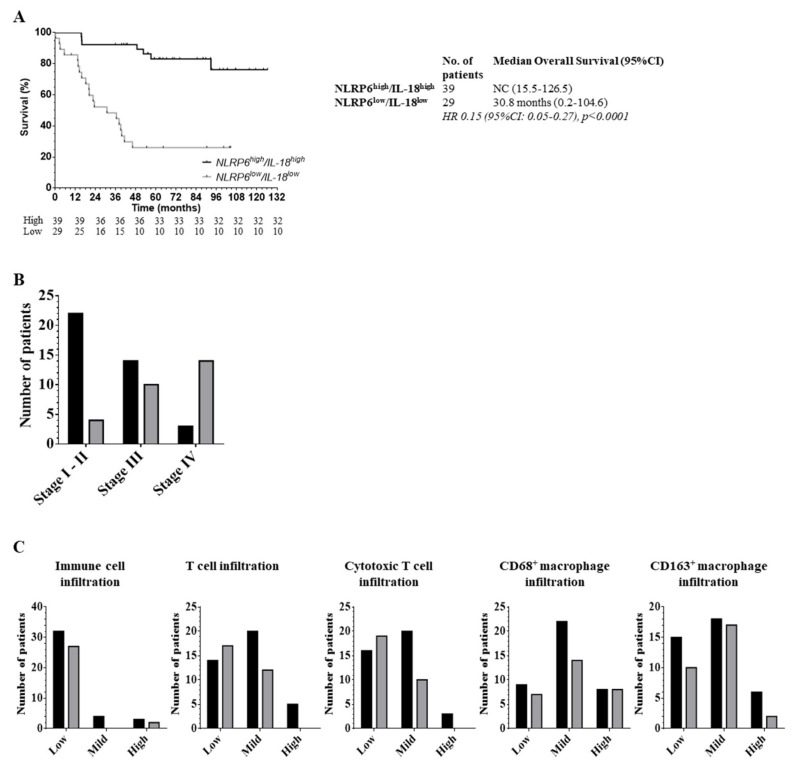
Downregulation of epithelial NLRP6/IL-18 is associated with a more advanced disease. (**A**) Overall survival of colorectal cancer patients based on NLRP6/IL-18 protein profile. NLRP6 and IL-18 were combined into a protein profile and segregated into NLRP6high/IL-18high and NLRP6low/IL-18low according to cut-off determined by ROC curves. Its impact on patient outcome was assessed by the comparison between NLRP6high/IL-18high and NLRP6low/IL-18low subgroups. A log-rank test stratified according to protein expressions was used. 95%CI: 95% confidence interval; HR: Hazard Ratio. A log-rank test stratified according to protein expressions was used. NC: could not be calculated. (**B**) Tumor aggressiveness based on the co-expression of NLRP6/IL-18. Data are expressed as absolute number of patients for each stage. We compared stage I-II (locally advanced), stage III (regionally advanced) and stage IV (metastatic disease). Black: NLRP6high/IL-18high; grey: NLRP6low/IL-18low. A χ2 test was used for the comparison. (**C**) Correlation between immune cells, T lymphocytes, CD8+ T cells, CD68+ or CD163+ macrophage infiltrates and co-expression of NLRP6/IL18 in the protein profile. Data are expressed as absolute number of patients for each immune cell infiltrate. Black: NLRP6high/IL-18high; grey: NLRP6low/IL-18low. A χ2 test was used for the comparisons.

Based on the crucial role of NLRP6 and IL-18 in colon physiology, we combined these markers into an epithelial inflammasome protein profile and found a strong correlation between patient outcome and epithelial co-expression of NLRP6 and IL-18 (Figure 5A). Indeed, while the 5-year survival rates were 46% and 42% for the NLRP6low and IL-18low tumors, respectively (Figure 2A), it only reached 26% for the NLRP6low/IL-18low tumors, with an 85% higher risk of death. Furthermore, patients whose tumors downregulated both biomarkers harbored a more metastatic disease (*p* < 0.0001) (Figure 5B). We observed no difference in macrophage infiltration using the NLRP6/IL-18 protein profile (Figure 5C). However, the number of NLRP6 high/IL-18high patients with mild and high CD8+ T cell infiltrate was more important than NLRP6low/IL-18low patients. The combined downregulation by epithelial tumor cells of NLRP6 and IL-18 is associated with a more advanced pattern and a worse outcome in CRC disease, potentially explained by a lower T cell infiltration.

## 3. Discussion

Inflammasome proteins are critical mediators of the host innate immune response to cell stress and infection. Recent data emphasize their role in gut homeostasis and cancer development from the early stages of disease all the way to advanced metastasis (for review see Reference [9]). Another level of complexity is that their expression and role in cancer pathophysiology vary upon organs, cell types studied in the tumor microenvironment or disease stage, leading to diverging data. Most data on inflammasomes are provided from mice or in vitro models and there is a crucial need to better understand their role in tumorigenesis. Furthermore, in human, inflammasome expression in cancer was only reported in small cohorts of patients at the transcriptomic level in bulk tumors. Therefore, we wanted to assess more widely the inflammasome expression in human colorectal cancer and its correlation to clinicopathological and immune infiltration parameters. To our knowledge, this is the first and largest evaluation of epithelial inflammasome proteins in human colorectal cancer and the first report of human NLRs as biomarkers of survival and cancer progression. Indeed, we found that colorectal epithelial cancer cells strongly downregulate all of the components of the inflammasome pathway at the protein level. Expression of some inflammasome components were also found decreased in the literature at the gene expression level but the mechanism of their loss was not well identified because little is known on the regulation of inflammasome gene expression. Expression of the ASC adaptor was found decreased due to hypermethylation in different models (glioblastoma, lung, breast, melanoma, prostate) [12]. Furthermore, through transcriptome analyses of bulk tumors from public databases, Liu et al. found three different patterns of inflammasome expression in colorectal cancer: a downregulation for NLRP1, NLRP3, NLRC4 and AIM2, an upregulation for NOD1 and NOD2 and no variation for NLRC5, NLRP6 and NLRP12 [8]. The downregulation by tumor cells of inflammasome components favors immune escape, by limiting the recruitment and activation of antitumor immune cells in a caspase-dependent manner. Furthermore, it was shown that some NLRs may display antitumor function in a caspase-independent manner as shown for AIM2 and NLRP12 [10,13].

Interestingly, among all the components investigated, only the downregulation of NLRP6, caspase-1 or IL-18 in epithelial cancer cells was correlated with an increased risk of death. Furthermore, we identified a protein profile, consisting of NLRP6 and IL-18 as a strong predictor of patient outcome, with a poor prognosis in the case of a low protein profile. 

This survival effect was probably partly due to a more advanced disease in NLRP6low and/or IL-18low tumors. Our protein profile did not include caspase-1 whereas it had a survival impact alone, because its association to NLRP6/IL-18 had no further effect on predictiveness. IL-18 is an important cytokine involved in colon physiology and mucosal immunity. IL-18 is involved in homeostatic renewal of the gut epithelial barrier, to prevent colitis. Indeed, IL-18-/- mice show an increased incidence of polyps, colitis and colorectal cancers [14], therefore illustrating its role in cancer protection under steady state conditions. 

IL-18 explains the anticancer effect of inflammasomes in a caspase-dependent manner, inducing the regulation of epithelial cell proliferation, the recruitment of immune cells and polarizing them towards developing cytotoxic function. Indeed, in IL-18-/- mice, as in our cohort, a lower infiltration of immune cells, leading to lower interferon-γ production was observed [15]. Interferon-γ is an important cytokine allowing the recruitment and activation of antitumor immune cells, which can direct antitumor immunity [16]. Cell-type specificity of IL-18 expression seems to confer different functional roles of this cytokine depending on its location in the tumor microenvironment. In our study, we found that stromal IL-18 expression had a more important impact on the immune lymphoid infiltrate, whereas epithelial IL-18 expression was strongly associated with disease evolution and patient outcome. This potent effect of IL-18 on patient survival is likely due a combination of non-redundant events including 1- tumor infiltration of T cells through stromal expression and 2- restricted epithelial events leading to the maintenance of the gut barrier including mucin production, tight junction maintenance and reduction of the stemness of colon epithelial cells (as reviewed in Reference [17]). These gut epithelial restricted events mediated by IL-18 are consistent with activation of the NLRP6 inflammasome in enterocytes [18]. We found that loss of IL-18 epithelial expression was correlated with a more advanced disease, which is consistent with data obtained from mouse models. Indeed, in a mouse model of liver metastasis from colorectal cancers, NLRP3-/- mice showed reduced IL-18 expression and this was associated with increased liver metastasis [19]. Rescue with exogenous IL-18 led to limitation of the metastatic process. This was due to decreased infiltration of Th1 and NK cells, critical cell types that mediate potent antitumor immune response [20]. These immune cells were also unable to confer tumor lysis (through downregulation of the Fas pathway). This was also reported in other tumor models such as melanoma [21]. We did not assess NK cell infiltration in our cohort but as reported in the literature, we also found less T cell infiltration in IL-18 low tumors T cell infiltration in tumors downregulating IL-18. Importantly, IL-18 production is one of the few cytokines lost rapidly during progression of lung cancer in patients as identified by deep immunophenotyping recently [22]. We did not find any clinical correlations with IL-1β, which is abundantly expressed in the tumor microenvironment of several cancers [23]. Mouse models have reported a protumoral role on proliferation, metastatic process [24], angiogenesis [25] and immunosuppression [26]. However, it has a controversial role according to tumor type and to compartment of secretion. This could explain the absence of prognostic role of this cytokine. 

In contrast to IL-18, we found no correlation between NLRP6 epithelial expression and immune infiltration. Thus, NLRP6 could have an antitumor role in a caspase-dependent manner through the secretion of IL-18 and in a caspase-independent manner as well. Indeed, in NLRP6-/- mice, decreased IL-18 secretion as well as maturation of functional immune cells was observed [27]. This led to a decreased immune cell recruitment and favored proinflammatory cytokine secretion such as TNFα and IL-6. NLRP6 can also regulate mucus secretion through the regulation of MUC2 [28]. Indeed, the loss of NLRP6 favors colitis through microbiome dysregulation and a defect in the mucus protective function [29,30]. It was evidenced in NLRP6-/- mice that cross-regulation between NLRP6 and the intestinal microbiome occurs through AntiMicrobial Peptide (AMP) production, as NLRP6 deficient mice favor dysbiosis [31]. The correlation between NLRP6 and tumor evolution could be explained by NLRP6-dependent regulation of some genes involved in the epithelial to mesenchymal transition, such as Wnt and Notch [32] or the cell-intrinsic regulation of cancer cell proliferation and migration. Finally, its role in mediating Type-I IFN signaling (as shown in virally-infected cells [33]) might trigger cell death of cancer cells in an inflammasome-independent manner.

In our work, we found an impact of NLRP6 and IL-18 expression on patient outcome, which highlights non-redundant roles in protection and survival. Indeed, NLRP6 and IL-18 are strongly involved in the regulation of the microbiome, which is now recognized as a critical player in colon inflammation favoring cancer. Cohousing of mice that were unable to produce IL-18 with normal mice limited colitis in knock-out mice, underlying an important role of microbiome dysbiosis in inflammation-induced colorectal cancer [34]. 

We assessed expression of inflammasome components in intestinal epithelial cells with the generation of a cytokeratin mask, to avoid contamination with stromal immune cells that also express inflammasome components. We focused on the epithelium because the literature has reported a diverging effect of inflammasomes according to tissue compartments. Indeed, in NLRP1-/- mice in hematopoietic or non-hematopoietic cells, it was shown that epithelial but not immune, expression of NLRP1 limited tumorigenesis [34]. Furthermore, the cellular compartment and the timing of IL-18 secretion in disease evolution seems to have a different impact on tumorigenesis [35]. It was shown in mouse models that gut epithelial IL-18 favored colitis and inflammation-induced cancer, whereas it was protective in established cancer. However, in our cohort, we found the same effect of IL-18 on patient survival either from epithelial or stromal cells but the effect was less important in stromal cell thereby underlying a potential role of the cell type of origin.

About 17% of colorectal cancers are associated with microsatellite instability in France. In our cohort, we found that stromal IL-18 was higher in MSI tumors patients. This is probably due to higher mutational burden of these specific subset of tumors, which are called “hot tumors” and are known to better respond to immune checkpoint inhibitors [36]. In all colorectal tumors, we found a correlation between high IL-18 expression and high T cell infiltration, most likely because IL-18 is one of the most important immune cell-recruiting cytokines. There was also a higher expression of PD-1 on immune cells that is consistent with their activation status. In most cases, established solid tumors create an immunosuppressive microenvironment among which the upregulation of PD-1 or sustained PD-L1 signaling in T cells after activation alter the cytotoxic response against the tumor. IL-18 is a major cytokine triggering T cell cytotoxicity towards Th1 polarization by inducing release of IFNγ and cytotoxic makers Granzyme/perforins from T cells and NK cell. Because of this relationship between IL-18 and PD-1 expression, participating to the immunosuppressive contexture, maybe IL-18 expression level could serve as a biomarker to help clinicians better stratify patients likely to respond to PD1-targeted immunotherapies. Furthermore, we found a correlation between IL-18 protein expression and immune infiltration, meaning that activation of NLR inflammasome pathways to mediate IL-18 secretion, could improve immunotherapy responses by turning cold tumors into hot known to have better response rates to current immunotherapies. Along this strategy, a NLRP3 agonist has been recently developed by BMS/IFM Therapeutics and is currently under clinical investigation for lung malignancies. Therefore, levels of NLR inflammasomes might serve clinicians either as biomarkers for immunotherapy response or as new therapeutic targets to prime and enhance antitumor immune responses through T cells and/or NK cells.

Our work had several limitations. First of all, it is a retrospective study made on a unique cohort within France and needs validation outside of the study area. Secondly, it is necessary to prospectively confirm our results on a larger cohort. Currently, we are assessing our protein profile in a new cohort of CRC patients, to validate these findings. Thirdly, this study does not allow us to assess the cellular mechanisms underscoring the strong NLRP6 and IL-18 prognostic role. More in vitro and in vivo studies are needed to better understood these results. 

## 4. Materials and Methods 

### 4.1. Tissue Micro-Array (TMA) Constitution

One hundred and four patients treated for a primary CRC at the Bergonié Institute (Bordeaux, France) between May 2008 and January 2013 were enrolled. Histology samples were obtained from surgery or biopsies and tissues were fixed in formalin and then paraffin-embedded. Clinical data were retrospectively collected from patient medical charts with special focus on age, gender, date of diagnosis and tumor-node-metastasis (TNM) stage. Histological type was determined as well as tumor grade and mutational status (Microsatellite Instability (MSI), KRAS and BRAF mutations).

Tissue cores with a diameter of 0.6 mm were removed from fixed paraffin-embedded tissue blocks and arrayed on a recipient paraffin block using a tissue arrayer (Beecher Instruments Tissue Arrayer, Sun Prairie, WI, USA) at the Molecular Biology Department of Bergonié Institute. Each tumor sample was punched in triplicate, along with 2 cores of matched normal mucosal tissue punched far away from the tumor. 

### 4.2. Fluorescent Tissue Staining

Immunofluorescent analysis was performed on a 5-µm fixed paraffin-embedded TMA section mounted on a charged slide. Slides were dewaxed on heating plate at 58°C for 10 min and in series of xylene and ethanol baths. Heat-induced epitope retrieval was done in target retrieval pH6 or pH9 buffer (Dako^®^, Glostrup, Denmark) according to the primary antibody for 20 min using a microwave oven. Slides were then blocked using 5% BSA for 10 min. Primary antibodies were incubated for predetermined duration at room temperature in antibody diluent (Dako^®^, Glostrup, Denmark), as indicated in Table S1. Slides were then washed three times in PBS 1X. Secondary antibodies [goat anti-rabbit (Alexa Fluor 594 nm), goat anti-mouse (Alexa Fluor 488 nm) and donkey anti-goat (Alexa Fluor 594 nm), all from ThermoFisher^®^ (Waltham, MA, USA)] were diluted (1:400) in antibody diluent and incubated for 1 h at room temperature in the dark. Sections were washed with PBS 1X three times and incubated with 4’,6-diamidino-2-phenylindole (DAPI) (2 μg/mL) for 10 min in the dark. Finally, slides were mounted with Fluoromount-G (Clinisciences^®^, Nanterre, France) and dried overnight at 4 °C in the dark. For each antibody, specificity was checked by western-blot or immunofluorescence assay on overexpressing cell lines fixed in formalin and paraffin-embedded.

### 4.3. Slides Acquisition (Figure S1)

Slides acquisition is detailed in the Supplementary Methods. Briefly, we used the slide scanner NanoZoomer from Hamamatsu. Cores were localized on TMA slides and slices were acquired using different fluorescent channels to detect cytokeratin, nuclei (DAPI) and inflammasome markers. Then, a cytokeratin mask (cellular segmentation) and a DAPI mask (nuclei segmentation) were performed and masks were superposed on inflammasome marker. Fluorescence corresponding to inflammasome expression was evaluated in cytokeratin-positive and DAPI-positive cells. Images were reviewed with NDP.View 2 (Hamamastu photonics Inc. Shizuoka Pref., Japan).

### 4.4. Immunohistochemistry

Immunohistochemical analysis was performed in all cases on 3 µm-thick serial sections from a representative formalin-fixed, paraffin-embedded (FFPE) block. We used the following antibodies: CD3 (clone 2GV6, prediluted, Roche^®^ Diagnostics), CD8 (clone C8/144B, dilution 1:25, Dako^®^, Glostrup, Denmark), CD68 (clone PG-M1, dilution 1:50, Dako^®^, Glostrup, Denmark), CD163 (clone 10D6, dilution 1/100, Leica^®^ Novocastra Laboratories, Wetzlar, Germany), PD-1 (clone NAT105, prediluted, Roche^®^ Diagnostics, Bâle, Switzerland) and PD-L1 (clone SP263, prediluted, Roche^®^ Diagnostics, Bâle, Switzerland). In 32 cases, heterologous differentiation was suspected and additional antibodies against desmin (clone D33, dilution 1:100, Dako^®^, Glostrup, Denmark), h-caldesmon (clone h-CD, dilution 1:50, Dako^®^, Glostrup, Denmark) and myogenin (clone LO26, dilution 1:20, Novocastra^®^, New Castle upon Tyne, United Kingdom) were used. After microwave oven heating (20 min in 0.1 M citrate buffer at pH 6), sections were incubated with biotinylated link antibody, with peroxidase-labelled streptavidin (LSAB™ + Kit; Dako^®^, Glostrup, Denmark) and then with diaminobenzidine solution (DAB; Dako^®^, Glostrup, Denmark). Omitting the specific primary antibody was used as negative controls. The tumor PD-L1 expression was considered positive if at least 1% of tumor cells were stained and PD-1 status was assessed on immune cells and considered positive if ≥ 1 %. Finally, CD3, CD4, CD8, CD68 and CD163 were assessed as low, moderate and high expressions by an expert pathologist.

### 4.5. Statistical Analysis

The samples were collected in accordance to French legislation and all patients gave their consent for the use of their biological samples in research. For cut-off determination for our markers, clinical and biological measurements were expressed as mean ( + /- standard deviation) or median (range) for continuous variables or as a number (percent) for categorical variables. Receiver-operating characteristics (ROC) curves and area under the curve were computed to assess the effectiveness of NLRC4 and NLRP6 to predict death (Figure S4). For age, patients were classified in 3 subgroups according to tertials. Statistical significance was set at *p* < 0.05. Data analysis was performed using the STATA 13.1 software, (StataCorpLP, College Station, TX, USA).

Overall survival (OS) was analyzed from the inclusion time to the death of patient. A Cox model was applied for survival analysis. Paired data were analyzed with a paired t test (parametric) or a Wilcoxon test (non-parametric). Associations between quantitative and qualitative variables were analyzed using t-test (parametric) or Mann Whitney (non-parametric) for 2 variables and one way ANOVA (parametric) or Kruskal-Wallis (non-parametric) for up to 2 variables. Univariate analyses were performed with χ2 or Fisher’s exact test. Statistics were performed using GraphPad Prism version 7.00 for Mac, GraphPad Software (San Diego, CA, USA). Results were considered significant if the *p*-value was <0.05.

## 5. Conclusions

In summary, we report here the first large evaluation of inflammasome components expression at the protein level and found that NLRP6 and IL-18, alone or in combination, were a strong predictor of patient outcome in colorectal disease. Their expression was strongly correlated to tumor evolution, meaning that their downregulation is associated with the metastatic process. Our study may help in better stratifying patients for prognosis in CRC. These results have to be confirmed on an external validation cohort. Furthermore, our protein profile could help clinicians in determining which patients may benefit from current. It would be of interest to better understand the mechanisms underlying downregulation of inflammasome components in the context of carcinogenesis and to determine which targets are regulated by these components. Finally, finding new ways to harness the innate immunity of tumors is currently of particular interest [32] and re-establishing normal expression of NLRP6 in tumor cells may confer therapeutic benefits by blocking progression towards advanced CRC.

## Figures and Tables

**Table 1:** Demographic and clinical features of colorectal cancer patients.

Characteristic		*N*	%
Gender	Male	60	57.7
	Female	44	42.3
Median age (years [range])		71.7 [38.6–88.4]	
Stage	I	8	7.7
	II	36	34.6
	III	37	35.6
	IV	23	22.1
Grade	1	8	7.7
	1-2	9	8.7
	2	76	73.1
	3	8	7.7
Mutational status	KRAS	19	18.3
	BRAF	4	3.8
MMR status	MSS	88	84.6
	MSI	16	15.4
5 years overall survival		64.4%	

## Supplementary Materials

The following are available online at https://www.mdpi.com/2072-6694/12/12/3500/s1, Figure S1. Image processing used to monitor the expression of epithelial inflammasome components; Figure S2: Correlation between epithelial expression ofNLRP1, NLRP3, AIM2, ASC, caspase-1 and IL-1B and immune infiltration; Figure S3. A: Correlation between stromal expression of NLRP6 or IL-18 and TNM stage and MSI status; B: Correlation between stromal expression of NLRP6 or IL-18 and immune infiltration assessed by immunohistochemistry for T cell lymphocytes (CD3+ and CD8+) and macrophages (CD68+ and CD163; Figure S4: ROC curves for cut-off determination for epithelial expressions of NLRP6 and IL-18. Table S1. List of antibodies used to stain paraffin-embedded tissues.

## Abbreviations

AMP	antimicrobial peptides
CRC	colorectal cancer
DAMP	danger-associated molecular patterns
DAPI	4’,6-diamidino-2-phenylindole
FFPE	formalin-fixed, paraffin-embedded
IFN	interferon
MSI	microsatellite instability
NLR	nucleotide-binding domain and leucine-rich repeat proteins
OS	overall survival
PAMP	pathogen-associated molecular patterns
PRR	pattern-recognition receptors
ROC	receiver-operating characteristics
TMA	tissue micro-array
TNM	tumor-node-metastasis
