# Supplementary material for: Prognostic Role of Inflammasome Components in Human Colorectal Cancer
Source: Cancers (Basel). 2020 Nov 24;12(12):3500. doi: 10.3390/cancers12123500 (PMC7760257; doi:10.3390/cancers12123500)
Supplement: Supplementary file 1 [file cancers-12-03500-s001.pdf]

Supplementary Materials: Prognostic Role of Inflammasome Components in Human Colorectal Cancer

C. Domblides, I. Soubeyran, L. Lartigue, I. Mahouche, F. Lefort, V. Velasco, T. Barnetche, P. Blanco, J. Déchanet-Merville and B. Faustin

Supplementary methods

Slides acquisition (Supplementary Figure 1)

Image handling

Images of the TMA are generated using the slide scanner NanoZoomer from Hamamatsu. Images are stored in NDPI image files. Homemade software was designed to perform the processing adapted to the study.

First slide image is display on the screen at low resolution. User select the 4 cores of at the extremity of the TMA to pre-localize the core grid. The localization of cores is improved by searching high intensity cores around each node of the grid. Localization of all cores are displayed in the screen to let user refine cores that would be poorly located.

Image of high resolution (20x) is exported for each core in TIF format and is named according to their position in the grid (A01, A02, …, B01, …)

Cytokeratin detection

Slides are cut sequentially, and successive slices are stained using different fluorescent biomarkers. One of those biomarkers stains Cytokeratin to reveal epithelium structures. Epithelium is detected using a simple threshold intensity on the cytokeratin channel followed by a closing operation to fill up small holes in the segmented region. The obtained region can be used to distinguish Epithelium structure from the rest on the image. It also can be transferred to a consecutive slice to reveal the position of the Epithelium in the consecutive slides.

Cytokeratin region transfer

To be able to transfer the Epithelium region to a consecutive slide, nuclei channel of a core image is register to nuclei channel in the consecutive slide. Nuclei repartition and density contains enough information to estimate the rotation and translation need to register the first image on the second one. The estimate rotation and translation are applied the full core image and the epithelium region mask. Epithelium region mask is now precisely aligned to the consecutive slice.

Cell detection

Nuclei DAPI channel is used to drive cell segmentation. Nuclei are detection using band pass follow by thresholding above the background. Nuclei clusters are spited using watershed strategy in order to obtain well separated nuclei segmentation. Cell segmentation is obtained by defining rings around nuclei. Rings of fixed width define the cytosolic area around nuclei.

Feature measurements

For each cell, intensity features are measured from fluorescent channels using maximum or average operator. Measurements are extracted for the different subcellular compartments (cells, nuclei and cytosol). Cell statistics are stored into csv files for further analysis.

Core alignment

Consecutive TMA slices are extracted from single paraffin blocks. Different biomarkers are stained from consecutive slices. In order to perform correlative analysis between the different channel, a method based on image registration where developed and used. For each core, image registration based on DAPI channel where applied. DAPI channel is used because it is the channel which is the most conserved from on slide to another. The image registration consists in finding the best rigid transform (translation + rotation) that allows to fit the first image into the second. This best transform is applied to all channel of the first slide. A new multiplexed image is obtained combining all channels of the first slice and all channels of the second slide. This multiplexed image is used to apply Cytokeratin mask detection to consecutive images.

Cytokeratin mask detection

The cytokeratin mask is obtained by processing cytokeratin channel using direct thresholding followed by morph math operations (opening and closing). This mask is used as a channel such as a feature express whether the cell is in or out the cytokeratin mask.

Cell positivity

Cell positivity is obtained by placing one or several thresholds on selected features. For example, we have counted the number of cells that are positive for a certain biomarker and inside the cytokeratin mask. The positive cells where displayed on the screen such as the user can control and finetune the threshold. After staining, slides were then digitalized with the Hamamatsu Nanozoomer 2.0HT scanner in collaboration with the Bordeaux Imaging Center (BIC). Imaging acquisition and fluorescence quantification was made by QuantaCell Inc. (Pessac, France). After computer-assisted image calibration, immunofluorescence quantification was obtained by measuring the fluorescence of each pixel in a DAPI-positive cell, calculating the median of all pixels in each cell and then assessing the mean of median intensity of all cells for each spot. Studied cells were identified with DAPI staining and epithelial cells (in normal and tumour tissues) with a cytokeratin mask. Indeed, quantification of inflammasome expression was done by applying a cytokeratin mask on each spot, to measure fluorescence of inflammasome markers only in cytokeratin/epithelial positive cells. Images were reviewed with NDP.View 2 (Hamamastu photonics Inc).

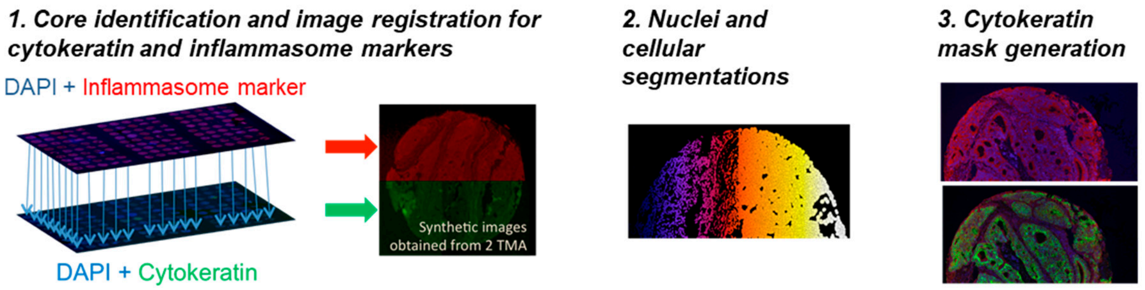

Figure S1. Image processing used to monitor the expression of epithelial inflammasome components. Slides were stained with antibodies targeting inflammasome components, cytokeratin for epithelial staining and 4′,6-diamidino-2-phenylindole (DAPI) for nuclear staining (**1**). Slides were digitalized with the Hamamatsu Nanozoomer 2.0HT scanner. A nuclear (DAPI positive) and a cellular segmentations were performed (**2**). A cytokeratin mask was then created and superimposed to inflammasome staining (**3**). Immunofluorescent expression quantification was obtained by measuring the cell intensity of DAPI positive cells in the cytokeratin mask on each spot.

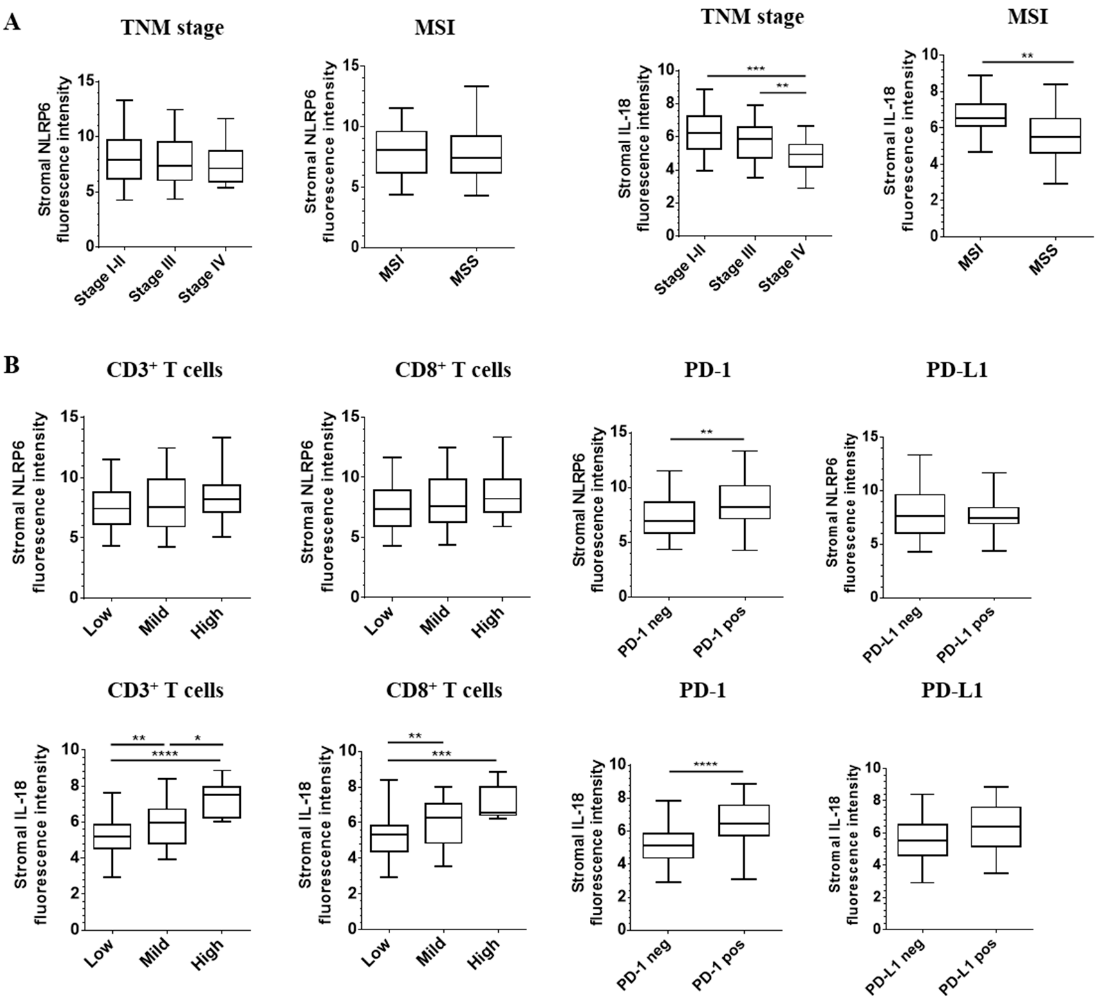

Figure S2. Downregulation of stromal IL-18 expression is associated with a more advanced disease and with a colder tumor. (**A**) Correlation between tumor stage and NLRP6 or IL-18 stromal expressions. For each protein, we assessed the correlation between fluorescence intensity and tumor stages, classified in stage I-II (locally advanced), stage III (regionally advanced) and stage IV (metastatic disease). A one-way ANOVA (parametric) or Kruskal-Wallis (non-parametric) tests were used to evaluate the significance of the differential expression between each disease stage. *P<0.05, **P<0.01, ***P<0.001 and ****P<0.0001. (**B**) Correlation between immune parameters and NLRP6 or IL-18 stromal expressions. For each protein, we assessed the correlation between fluorescence intensity of inflammasome component and immune infiltration assessed by immunohistochemistry for T cell lymphocytes (CD3+ and CD8+). We also evaluated PD-1 and PD-L1 expressions. A one-way ANOVA (parametric) or a Kruskal-Wallis test (non-parametric) were used to evaluate the correlation between expression intensity and immune infiltrate levels. *P<0.05, **P<0.01, ***P<0.001 and ****P<0.0001.

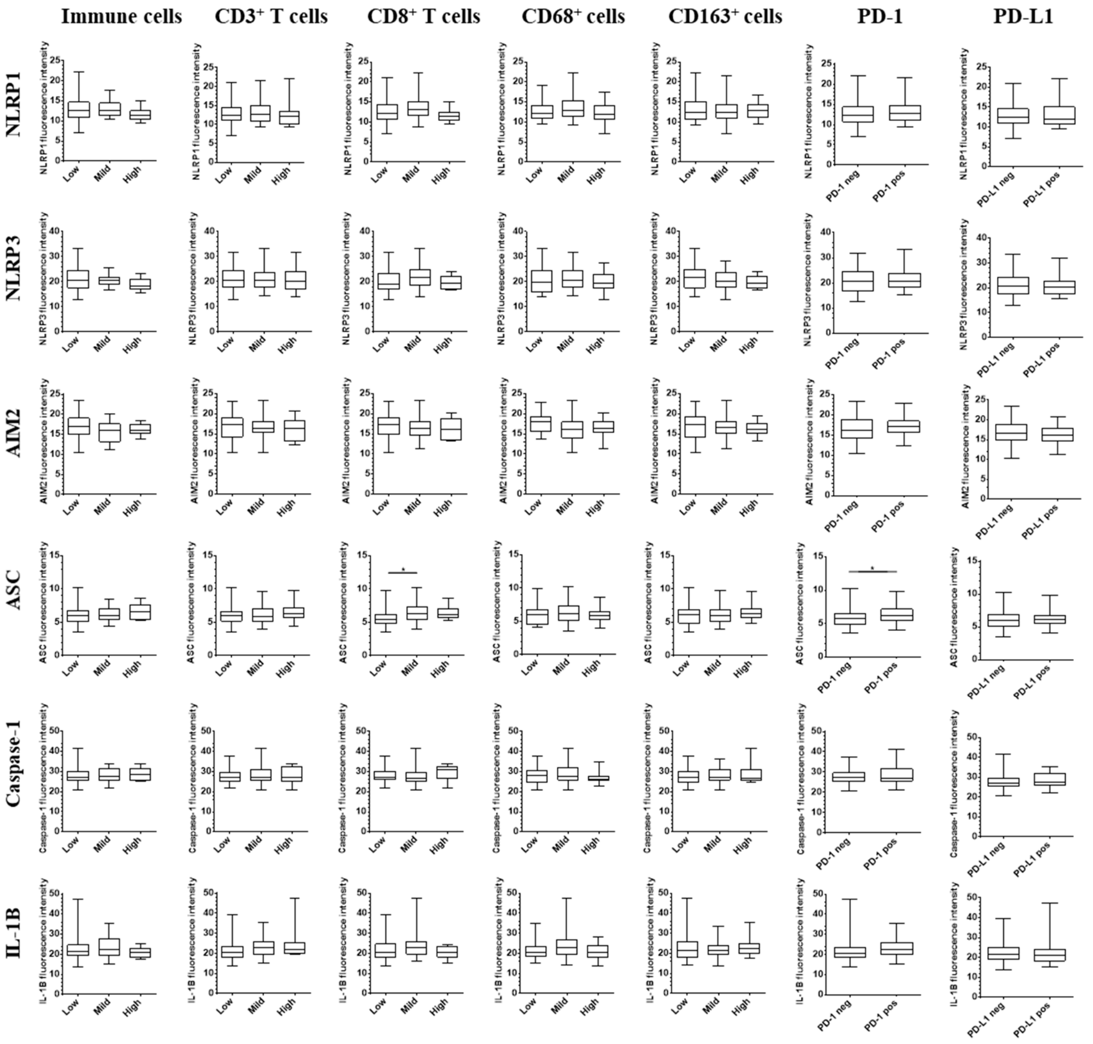

Figure S3. (A): Correlation between stromal expression of NLRP6 or IL-18 and TNM stage and MSI status. For each protein, we assessed the correlation between fluorescence intensity and tumour stages, classified in stage I-II (locally advanced), stage III (regionally advanced) and stage IV (metastatic disease). A one-way ANOVA (parametric) or Kruskal-Wallis (unparametric) tests were used to evaluate the significance of the differential expression between each disease stage. **<P0.01 and ***P<0.001. (**B**)**:** Correlation between stromal expression of NLRP6 or IL-18 and immune infiltration assessed by immunohistochemistry for T cell lymphocytes (CD3+ and CD8+) and macrophages (CD68+ and CD163+). We also evaluated PD-1 and PD-L1 expressions. A one-way ANOVA (parametric) or a Kruskal-Wallis test (unparametric) were used to evaluate the correlation between expression intensity and immune infiltrate levels. *P<0.05, **<P0.01, ***P<0.001 and ****P<0.0001.;.

NLRP6

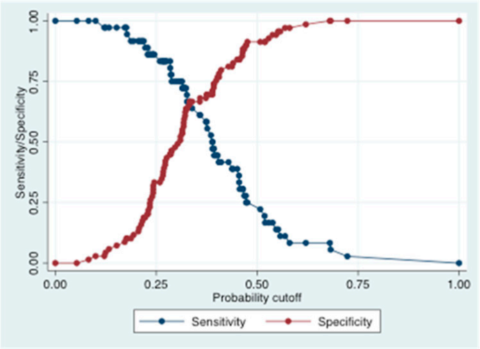

**Cut-off**	11.466567
**Sensibility**	72.2% [54.8% - 85.8%]$_{95\%}$
**Specificity**	63.8% [51.3% - 75%]$_{95\%}$

IL-18

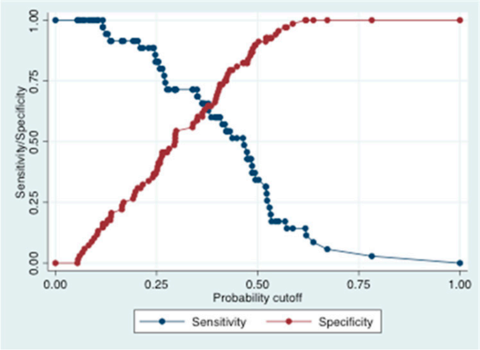

**Cut-off**	7.70055
**Sensibility**	60% [42.1% - 76.1%]$_{95\%}$
**Specificity**	73.5% [61.4% - 83.5%]$_{95\%}$

Figure S4. ROC curves for cut-off determination for epithelial expressions of NLRP6 and IL-18.

Table S1. List of antibodies used to stain paraffin-embedded tissues.

Target	Clone	pH	Dilution	Secondary
Cytokeratin	AE1-AE3	pH 6	1/50	Mouse
NLRP1	ALX-801-803	pH 6	1/300	Mouse
NLRP3	ALX-804-819	pH 6	1/250	Mouse
NLRP6	ABF29	pH 6	1/500	Rabbit
AIM2	OAEB01074	pH 9	1/50	Goat
Caspase-1 (p10)	LS-C312683	pH 9	1/500	Rabbit
ASC	HPA049074	pH 6	1/100	Rabbit
IL-1β	3A6	pH 6	1/100	Mouse
IL-18	HPA003980	pH 6	1/200	Rabbit
